# Osmolar-gap in the setting of metformin-associated lactic acidosis: Case report and a literature review highlighting an apparently unusual association

**DOI:** 10.1097/MD.0000000000022492

**Published:** 2020-10-09

**Authors:** Mohamed Nabil Elshafei, Mohammed Alamin, Mouhand F.H. Mohamed

**Affiliations:** a Internal Medicine Department; b Clinical Pharmacy Department, Hamad General Hospital, Hamad Medical Corporation, Doha, Qatar.

**Keywords:** case report, MALA, metformin acidosis, metformin toxicity, osmolal gap, osmolar gap

## Abstract

**Rationale:**

Metformin-associated lactic acidosis (MALA) is a rare adverse effect that has significant morbidity and mortality. MALA is a high anion gap (AG), nonosmolar acidosis. Associated osmolar-gap (OG) is rarely reported, so finding an OG may make the diagnosis of MALA challenging.

**Patient concerns:**

Forty-five years’ old type II diabetic patient on metformin presented to emergency with a two-day history of vomiting, watery diarrhea, and mild abdominal discomfort. On examinations, he looked dehydrated. Investigation revealed acute kidney injury (AKI) with a high lactic acid (LA) level of 24 mmol/L, pH of 6.8, AG of 40, and an OG of 20 mOsm/kg

**Diagnoses:**

The presence of an OG made the diagnosis challenging; the history was negative for alcohol, osmolar substance, or illicit drug ingestion or use. The toxicology screen was negative. After ruling out plausible causes of AG and OG, MALA was deemed the likely reason for his presentation likely precipitated by dehydration and AKI.

**Interventions:**

He underwent two sessions of hemodialysis, afterward managed with fluid hydration.

**Outcomes:**

On day 3, he was in the polyuric phase suggestive of acute tubular necrosis. His serum creatinine improved afterward with improved acidosis; after 8 days, he was discharged in stable condition.

**Lessons:**

MALA is a rare side effect of metformin therapy. Acute kidney injury is a known precipitant of MALA. In our review, we highlight the association of MALA and the presence of an OG. We believe that treating physicians should be aware of this relationship to avoid delaying or overlooking such an important diagnosis.

**Learning Points**

- Lactic acidosis is a rare complication of metformin therapy that needs timely identification and management.
- Very high lactic acid can lead to an osmolar gap. However, common causes of an osmolar-gap like ethanol ingestion and toxins are to be ruled out.
- Clinicians should counsel patients to seek medical advice when feeling sick while on metformin therapy.

## 1 Introduction

Metformin is an oral hypoglycemic agent that works by inhibiting hepatic gluconeogenesis.^[1]^ It is globally used as a first-line type 2 diabetes treatment,^[2]^ perhaps due to its effect on glucose reduction, weight neutrality, a favorable risk profile, and the low risk of hypoglycemia.^[3–5]^ Despite being effective and generally safe,^[6]^ metformin-associated lactic acidosis (MALA), a dreadful rare side effect, has always been a clinicians’ concern, especially when dealing with patients suffering from liver or kidney disease.^[7]^ MALA is a high anion-gap (AG) acidosis that is generally thought to be not associated with osmolar-gap (OG). Here we present a case of MALA associated with an OG highlighting this seemingly unusual association; we discuss the possible mechanism, management, and argue that OG maybe not an infrequent occurrence in patients with MALA.

## 2 Case description

We present the case of a 45-year-old gentleman, known diabetic for 6 years on metformin 1 g, twice a day, with reasonable control (hemoglobin A1c 6.4%). He was admitted to our emergency feeling unwell with 2 days’ history of vomiting and watery diarrhea, also associated crampy periumbilical pain. He denied a history of similar complaints in his companions, no change in his dietary habits preceding symptoms’ onset. He drinks alcohol occasionally; the last drink was 6 months before the presentation—no illicit drug use history. The initial vital signs were normal. Soon later, he became tachycardic with heart rate reaching 120 bpm, blood pressure 127/70 mmHg, and a normal temperature. He looked dehydrated; otherwise, his examination was unremarkable.

Laboratory workup was significant for initially low glucose 2.5 mmol/L (3.3–5.5 mmol/L), high serum creatinine 632 μmol/L (62–106 umol/L), and high blood urea nitrogen of 21 mmol/L (2.8–8 mmol/L). Serum electrolytes were as follows: potassium (K+) 6.3 mmol/L (3.5–5.1 mmol/L), sodium (NA) 144 mmol/L (135–145mmol/L), bicarbonate (HCO3) 5.8 mmol/L (23–29 mmol/L), and serum chloride 99 mmol/L (99–107 mmol/L). Serum aminotransferases were normal (ALT 31 U/L, AST 27 U/L). He had significant high AG metabolic acidosis with PH of 6.8 (7.35–7.45) and AG of 40, lactic acid (LA) strikingly high reaching up to 24 mmol/L (0.5–2.2 mmol/L). With LA of 19 mmol/L, his OG measured 20 with serum osmolarity of 340 mmol/kg (275–295 mmol/kg). Toxicology screening, including serum ethanol, acetaminophen, salicylate, and urine for oxalate crystal, was negative.

**Figure 1:**
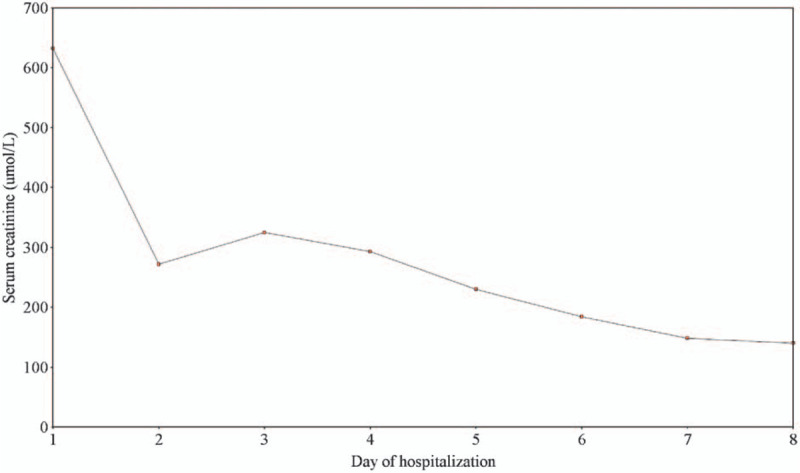
Serum creatinine trend during hospitalization.

**Figure 2:**
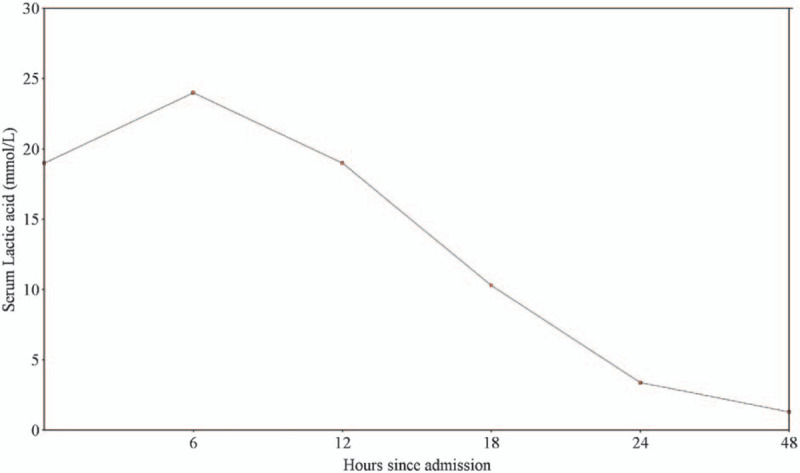
Serum lactic acid clearance during hospitalization.

Acidosis failed to respond to initial fluid hydration, including intravenous bicarbonate. Thus, the patient underwent 2 sessions of sustained low-efficiency hemodialysis (HD). After HD, his electrolyte disturbances improved, and intravenous fluid hydration continued. Acute kidney injury (AKI) workup, including antinuclear antibodies (ANA), antinuclear cytoplasmic antibodies, creatinine kinase level, and sepsis workup were unremarkable. Starting day 3, the patient was in a polyuric phase, suggestive of acute tubular necrosis (ATN), producing between 3 and 6 L per day, and his serum creatinine was decreasing. He was discharged after 8 days of hospitalization in good condition with a serum creatinine of 140 μmol/L (62–106 μmol/L). Figure 1 shows serum creatinine trend during the hospitalization, Figure 2 depicts serum lactic acid clearance during hospitalization.

Our patient had an AKI with significant lactic acidosis and an OG. Given the significant level of lactic acid, MALA was the likely diagnosis. Our patient was on metformin for a long time, and he did not attempt an overdose; hence, we think that MALA was likely precipitated by dehydration leading to prerenal insult and AKI. Eventually, his prerenal insult led to acute tubular necrosis, as evident by the polyuric phase the patient exhibited on day three.

## 3 Discussion

Metformin, an oral hypoglycemic agent, is considered the first-line therapy for type II diabetes mellitus.^[2,6]^ Metformin is generally effective and safe.^[6]^ The associated adverse event (AE) rate is estimated to be around 20% to 30%, with the majority of these AE in the form of gastrointestinal related symptoms, such as abdominal discomfort and nausea.^[6]^

In extreme cases, metformin can rarely lead to lactate build-up and, eventually, acidosis, a condition known as Biguanide or metformin-associated lactic acidosis (MALA). This condition (MALA) was first described in 1977,^[8]^ and since then, studies have emerged exploring this rare entity. The available data suggest that the pooled incidence averages around 3 to 10 per 100,000 person-years.^[9]^ Despite being rare, MALA is associated with significant morbidity and mortality that is as high as 61%.^[10]^ MALA presentation may vary with the presence of gastrointestinal symptoms, hemodynamic compromise, tachypnea, and abnormal mentation. This atypical nature may hinder or delay proper diagnosis and care.^[11]^

MALA is postulated to be due to the effect of metformin on transitioning the metabolism of glucose from aerobic to anaerobic, which leads to increased lactate levels^[12,13]^; however, in a therapeutic dose, this is likely to achieve a therapeutic effect without inducing relevant clinical acidosis.^[9]^ Therefore, MALA usually develops in the presence of conditions altering lactate metabolism, either increasing lactate production (metformin overdose), or impairing its clearance (hypo-perfusion, acute infection, renal or hepatic insufficiency).^[9]^ The term MALA has been debated and questioned by Lalau et al with the suggestion of differentiating between; metformin-unrelated lactic acidosis (MULA), metformin-induced lactic acidosis (MILA) and metformin-associated lactic acidosis, the details of differences and definitions of each condition are beyond the scope of our review. However, it is worthy to note that the author postulated that majority of the cases of MALA are MULA.^[9]^

Our case was interesting, not only because of the presence of the rare MALA entity, but also the additional finding of an OG. We wanted to explore the frequency of OG in patients with MALA. We systematically searched PubMed without limitations using the terms; (((osmolar gap) OR (increased Osmolarity)) OR (osmolal gap)) AND ((((MALA) OR (metformin-associated lactic acidosis)) OR (metformin toxicity)) OR (metformin acidosis)). Our search retrieved only 3 citations and they were all not relevant to our review question. It seems that the authors did not highlight this relationship in their cases or studies. A search in google scholar and free text on google retrieved 10 relevant citations^[14–23]^ out of which 9 cases described cases of MALA in which OG was present. It was highlighted by the authors in 4 cases discussing an extensive workup to rule out causes of the OG and even attempts to start Fomepizole till ruling out ethylene glycol toxicity. The highest OG reported was 89 mOsm/L. Table 1 shows the results of the literature review). Spiller et al. reviewed the Toxic Exposure Surveillance System (TESS) looking for metformin toxicity, in 9 fatalities related to metformin toxicity, 4 cases were found to have an elevated OG, and in all instances, ethylene glycol and methanol were negative.^[23]^

**Table 1:**
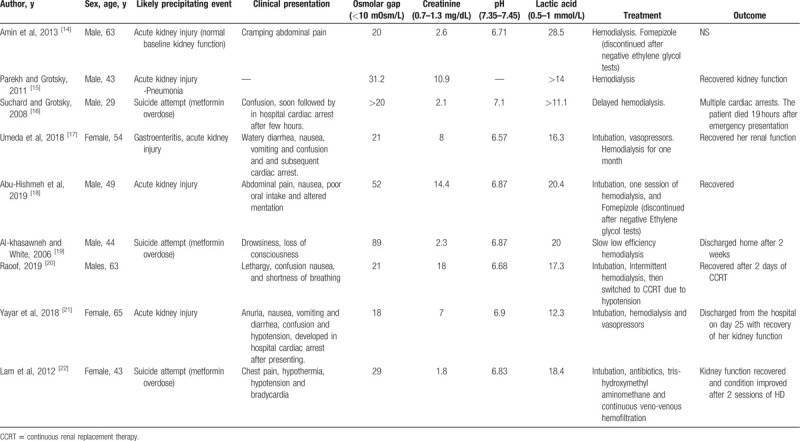
Summary of MALA cases associated with an osmolar-gap.

We reviewed the relationship between lactic acidosis and OG. We found an interesting study by Schelling et al^[24]^ exploring whether an elevated OG is specific to alcohol intoxication. In an arm including 23 patients with lactic acidosis, the OG was found to be high with a mean of 17 mOsm/L. The authors concluded that the OG is not specific for alcohol intoxication and that lactic acidosis can lead to an OG. However, the exact mechanism remains uncertain.

In the presence of an OG, the diagnosis of MALA may be challenging and even overlooked. However, clinicians must be aware that significant lactic acidosis can lead to an OG. We have highlighted in our review that severe cases of MALA specifically presented with an OG. Whether the OG in these cases is purely related to lactic acid build-up or due to metformin remains unclear; additionally, the prevalence of OG in MALA is unknown as not all cases, or studies reported OG measurements. We suggest when MALA is suspected in the event of an OG, that a quick workup to exclude other causes of an OG be performed, but that should not delay timely identification and treatment of MALA.

Our case is compelling because it is the first case highlighting the MALA association with an OG and discussing it in-depth with a systematic scoping search. Physicians should be aware of this possible relationship. Although rare, patients should be counseled on this side effect and asked to report to physicians when feeling sick.

## 4 Conclusion

MALA is a rare side effect of metformin. Nevertheless, associated with significant morbidity and mortality; thus, it should be diagnosed and treated promptly. High lactic acid and MALA can lead to an OG. After ruling out the common causes of an OG, the presence of an OG should not hinder a timely diagnosis and management of MALA. This relationship should be further studied. Patients on metformin therapy should be counseled about the risk of MALA and advised to seek medical advice when feeling sick.

## Acknowledgments

The authors thank Qatar National Library for funding the publication of this article. The authors acknowledge the efforts of the treating medical team, especially Dr. AL Mehdi Alrayyes. Dr. Zahid and Dr. Karim Bayomi.

## Author contributions

^∗^MFHM and MN contributed equally to this paper. MN and MFHM conceived the idea and wrote the initial draft. MN and MFHM constructed the table and figures. MFHM reviewed and updated the manuscript and supervised the work. All authors reviewed the final manuscript and approved it for publication.

## Abbreviations

Abbreviations: AG = anion gap, AKI = acute kidney injury, ALT = alanine aminotransferase, AST = aspartate aminotransferase, HD = hemodialysis, LA = lactic acid, MALA = metformin-associated lactic acidosis, OG = osmolar-gap.
